# The predictive role of intolerance of uncertainty and trait of worry in breast cancer patients: A prospective, observational, single-center clinical study

**DOI:** 10.3389/fpsyg.2023.1092060

**Published:** 2023-04-17

**Authors:** Malihe Shams, Susanna Pardini, Paola Del Bianco, Caterina Calabrese, Gian Luca De Salvo, Caterina Novara

**Affiliations:** ^1^Istituto Oncologico Veneto–IRCCS, Padua, Italy; ^2^Department of General Psychology, University of Padova, Padua, Italy

**Keywords:** breast cancer, intolerance of uncertainty, worry, longitudinal study, psychological assessment

## Abstract

**Background:**

Breast cancer diagnosis and treatment compromise well-being in a pervasive way, and negative consequences may remain after recovery. The psychological side of breast cancer has been extensively investigated; however, the role of intrusive thoughts and intolerance of uncertainty have been studied less systematically.

**Objectives:**

The present study aimed to prospectively evaluate worry content, depression, anxiety, and post-traumatic stress symptoms and to define the role of the trait of worry and intolerance of uncertainty (IU) related to breast cancer.

**Methods:**

Patients with their first breast cancer diagnosis were enrolled in a single-center, prospective observational trial. The trait of worry and IU were assessed using the Penn State Worry Questionnaire (PSWQ) and the Intolerance of Uncertainty Scale-Revised (IUS-R). The psychological aspects were evaluated using the Worry Domains Questionnaire (WDQ), the Beck Anxiety (BAI), Beck Depression Inventory-II (BDI-II), and the Impact of Event Scale-Revised (IES-R). Questionnaires were administered in a randomized sequence at diagnosis (T0), 3 months post-diagnosis (T1), and 12 months post-diagnosis (T2).

**Results:**

One hundred and fifty eligible patients were enrolled in the study and provided the T0 assessment. Further compliance rates were 57% at T1 and 64% at T2. All patients showed a significant and continuous increase in the IES-R scale (*p* < 0.0001) from diagnosis to the end of the study, while no significant changes were observed for the WDQ, BAI, and BDI-II scales. The clinical PSWQ levels and/or high levels of the IUS-R score were the only variables that aided the distinction between patients who maintain high levels of depression, anxiety, and post-traumatic disorders and those who did not.

**Conclusion:**

An early assessment of the components of the trait of worry and intolerance of uncertainty could be critical in identifying patients with a higher psychopathological risk. Furthermore, if future studies confirm the present findings, support and monitoring throughout the prognosis may present crucial benefits, and possibly affect the course of treatment.

## Introduction

Breast cancer is a pathology that can affect women at any age after puberty with an increased rate in later life. It is considered the most prevalent cancer globally, with 2.3 million women diagnosed in 2020 (WHO, 2021).

Breast cancer diagnosis presents challenges to the patient’s physical and psychological well-being (Dooley et al., 2017). Frequently, treatments deployed in order to manage the disease (e.g., radiotherapy, chemotherapy, hormonal, or antibody therapy) can negatively impact the quality of life even after recovery (Fong et al., 2012; Moo et al., 2018). Therefore, the end of treatment should be considered a critical moment in which the patient’s worries related to the actual or perceived loss of support from healthcare centers, and apprehension associated with the possibility of relapse are considered (Cappiello et al., 2007; Costanzo et al., 2007).

The psychological side of breast cancer has been extensively investigated, showing that anxiety, distress and depression are some of the more common psychological disorders experienced during the entire disease trajectory (e.g., Mertz et al., 2012; Lo-Fo-Wong et al., 2016; Ng et al., 2017; Gieseler et al., 2018; Guimond et al., 2019; Dinapoli et al., 2021).

The perceived sudden and catastrophic nature of a cancer diagnosis may promote the onset of Post-Traumatic Stress Disorder (PTSD) (Wu et al., 2016; Van Oers, 2019). Among women with breast cancer, PTSD prevalence varies considerably, up to 32.3% of the population. Heterogeneity could be related to the age at the moment of diagnosis, educational level, socioeconomic status, and the stage of diagnosis (Naidich and Motta, 2000; Wu et al., 2016; Brown et al., 2020). Moreover, there was no agreement between studies investigating the evolution of symptomatology over time. In this regard, to address the issue a literature review has highlighted four heterogeneous developmental trajectories of PTSD symptomatology (Van Oers, 2019): (1) a symptom reduction after diagnosis and during the treatment phase (Mehnert and Koch, 2007; Vin-Raviv et al., 2013; Bulotiene and Matuiziene, 2014; Voigt, 2016; Chan et al., 2018); (2) an increase of symptoms as a function of time (Cordova et al., 2017); (3) a symptom fluctuation during the first years after diagnosis (*ibidem*); (4) a presence of symptoms at a subclinical level that could be followed by a PTSD diagnosis (Utzon-Frank et al., 2014). Contrarily, research by Voigt (2016) showed that PTSD symptomatology has already been present at the diagnosis phase and that adjuvant and surgical therapies (such as mastectomy) did not promote symptomatology deterioration.

Recent literature has displayed that particular psychological trait constructs, such as Intolerance of Uncertainty (IU), can contribute to the presence of common psychological disorders (e.g., anxiety, depression, and post-traumatic disorders), both in people with and without a cancer diagnosis (e.g., Lerman et al., 1995; Mathews and MacLeod, 2002; Costa-Requena et al., 2011; Oglesby et al., 2016; Tan et al., 2016; Hill and Hamm, 2019; Romeo et al., 2022). Indeed, IU can be defined as a predictor and a maintenance factor of anxiety components already studied independently on cancer (Freeston et al., 1994; Dugas et al., 1997). It could be defined as a dispositional psychological construct characterized by a pervasive difficulty in tolerating aversive reactions to situations perceived as unclear or poorly controlled and negative beliefs on uncertainty (Buhr and Dugas, 2009; Bottesi et al., 2019).

More recent studies have shown a trans-diagnostic role of IU as a common factor in other psychopathologies besides anxiety disorders, such as depression and post-traumatic stress disorder (Einstein, 2014; Carleton, 2016a,b; Bottesi et al., 2019). The malleability of IU in various pathologies highlights the importance of its treatment by deploying a protocol compared to the mere focus on specific disorder features (McEvoy and Erceg-Hurn, 2016; Norton and Paulus, 2016).

Another important related trait construct to IU is Worry, defined as an excessive and uncontrollable concern about what could happen in the future that impacts different aspects of daily life (e.g., finances, relations, work). This construct is characterized by cognitive and emotional processes centered around the perception of uncontrollable fear and is focused on adverse outcomes involving a high level of selective attention on what is perceived as dangerous, usually connected to biased information processing aimed at managing the situation (Borkovec, 1994; Roemer et al., 1997; Craske and Tsao, 1999; Barlow, 2002; Borkovec et al., 2002). One crucial aspect of worry as a trait is its role in affecting distress in uncertainty by increasing a distorted perception of control over future situations (Greco and Roger, 2003). Like IU, the trait of worry has a pervasive impact characterizing anxiety and depressive disorders (Barlow, 1988; Molina and Borkovec, 1994; Dugas et al., 1998). In general, people that display high IU and worry features could experience a greater fear of cancer recurrence, and have distress, anxiety, post-traumatic and depressive symptoms when they are obliged to cope with uncertain situations such as waiting for medical results or the oncological disease prognosis (e.g., Shaha et al., 2008; Eisenberg et al., 2015; Cessna Palas et al., 2021). Demographic features could have a role in moderating the relationship between mental health and intolerance of uncertainty (e.g., Hill and Hamm, 2019).

Not many studies in the literature investigated the longitudinal course of psychological symptoms following a breast cancer diagnosis. A recent longitudinal study put in light how essential is investigate psychological symptomatology during the different phases of cancer from diagnosis to the follow-up after treatment, since it permits to understand the presence of risk and protective factors able in influencing the patients’ well-being (Romeo et al., 2020). This research investigated the relationship between depressive symptomatology, assessed at the time of diagnosis and 2 years later, and the Posttraumatic Growth, showing how positive psychological changes experienced after the struggle with cancer where higher in the group of patients that were no longer depressed rather than individuals that continued in experiencing depressive symptoms (Romeo et al., 2020).

To the best of our knowledge, the impact of IU and worry as a trait and predictive constructs on other common psychological problems have not been deeply longitudinally studied during different salient phases in a sample composed of individuals with breast cancer (Hill and Hamm, 2019). This type of analysis could be valuable to the identification and implementation of specific psychological rehabilitation protocols for breast cancer patients promoting overall well-being (Ramírez-Vélez et al., 2021).

The primary aim of this study was to prospectively evaluate worry content, depression, anxiety, and post-traumatic stress symptoms and to define whether the trait of worry and IU related to breast cancer were associated with high levels of psychopathological symptoms from diagnosis through treatment and follow-up. A secondary aim was to assess whether socio-demographics, clinical, and treatment variables influenced baseline trait components.

It has been expected that participants characterized by higher levels of worry, and IU present more anxiety, depression, and traumatic events than the others, independently from the assessment phase.

## Methods

The study was designed as a single-center, prospective, observational cohort investigation. Patients were eligible and included in the study if they had histologically proven diagnosis of primary breast carcinoma, were aged between 18 and 75, and were able to understand the investigational nature of the study. Patients were excluded if they: (1) could not adequately understand the Italian language, (2) had a psychological or psychopharmacological treatment in progress, (3) had a head injury, degenerative or cardiological disease, (4) previously received a cancer diagnosis other than breast carcinoma and have already undergone treatment. All eligible patients signed an Independent Ethics Committee/Institutional Review Board-approved written informed consent form before the baseline assessment. The current study was conducted following the Declaration of Helsinki and the protocol n. 642 identified by “IOV-BC-1-2015 IT&W” was approved by the IOV Ethical Committee on 12 December 2015.

Female patients who underwent primary surgery at the Veneto Institute of Oncology (Italy) were asked to complete a randomized sequence of questionnaires at diagnosis (T0), during treatment at 3 months post-diagnosis (T1), and during the follow-up at 12 months after diagnosis (T2). Each patient self-reported socio-demographic information about age, marital status, cohabitation, level of education, and current employment. Clinical data were also collected and included the type of surgery, stage of disease, and type of treatment planned.

Between November 2015 and March 2017, 150 eligible patients were enrolled in the study. All patients provided a baseline assessment at diagnosis, and further compliance rates over the study period were 57% (85 patients) at T1, and 64% (96 patients) at T2. Of 150 eligible patients at baseline, 55 completed the questionnaires at all 3-time points, and 24 completed only the baseline assessment. The main reason for missing questionnaires was the difficulty in intercepting and engaging the patients during treatment and follow-up visits.

### Measures

The IU was measured using the Intolerance of Uncertainty Scale-Revised (IUS-R; Freeston et al., 1994; Bottesi et al., 2015), a 12-items short form of the original version (Bottesi et al., 2019) that measures reactions to uncertainty, ambiguous situations, and the future (e.g., Uncertain events upset me greatly). This shorter version was used for the current study because the 27-item version has several items that appear to pertain specifically to GAD and might better account for symptoms of worry as a trait than those of other anxiety disorders (Carleton et al., 2010; Gentes and Ruscio, 2011). Participants rate each item on the IUS-R from 1 (Not at all characteristics of me) to 5 (Entirely characteristic of me), with total scores ranging from 12 to 60, and higher scores indicating greater IU. The measure consists of a total score and two subscales representing approach and avoidance responses to uncertainty, respectively (Birrell et al., 2011). The “Prospective IU” subscale measures the desire for predictability, preferences for knowing what the future holds, anxiety about future uncertain events, and active engagement in seeking information to increase certainty. The “Inhibitory IU” measures avoidance and paralysis in the face of uncertainty. The IUS-R total and subscale scores have good psychometric properties in both clinical and non-clinical samples (Carleton et al., 2007, 2012; Khawaja and Yu, 2010; McEvoy and Mahoney, 2011; Helsen et al., 2013; Jacoby et al., 2013) and internal consistency has also been confirmed for the current sample (0.82 < Cronbach’s α < 0.90; 0.66 < McDonald’s ω_h_ < 0.72; 0.87 < McDonald’s ω_t_ < 0.92) (Flora, 2020). For this study, we used the IUS-R total score. Considering the average and standard deviation obtained by a sample of the general Italian population (*M* = 26.73; SD = 8.20), patients were split into high and low IUS groups based on the current sample median of 27.

The Penn State Worry Questionnaire (PSWQ; Meyer et al., 1990; Morani et al., 1999) was used to assess the WCP; it has 16 items on a Likert scale from 1 to 5. Scores range from 16 to 80, with higher scores indicative of higher levels of trait worry. Eleven items are worded in the direction of pathological worry, with higher numbers indicating more worry (e.g., Once I start worrying, I cannot stop). In comparison, the remaining five items are worded to indicate that worry is not a problem, with higher numbers indicating less worry (e.g., I never worry about anything). The total score was split into high and low PSWQ groups based on the recommended cut-off score of 41. The higher PSWQ scores reflect greater levels of pathological worry. Its internal consistency was good (original version: Cronbach’s *α* = 0.93; Italian version *α* = 0.85). The current study highlighted excellent internal consistency (Cronbach’s *α* = 0.90; McDonald’s ω_h_ = 0.78; McDonald’s ω_t_ = 0.93) (Flora, 2020).

The Worry Domains Questionnaire (WDQ; Tallis et al., 1994; Morani et al., 1999) is a self-report questionnaire based on 25 items investigating worry content. The scoring is based on a Likert scale from 0 to 4. Higher scores indicate higher worry experienced about specific domains. The WDQ yields a total score, which is the sum of scores on five subscales titled (1) Relationships, (2) Lack of Confidence, (3) Aimless Future, (4) Work, and (5) Financial. Good internal consistency has been evidenced for the current sample (0.74 < Cronbach’s *α* < 0.93; 0.58 < McDonald’s ω_h_ < 0.79; 0.78 < McDonald’s ω_t_ < 0.94) (Flora, 2020).

The Beck Anxiety Inventory (BAI; Beck et al., 1988; Sica et al., 2009) is a 21-question multiple-choice self-report inventory used for measuring the severity of anxiety. The questions ask about common symptoms of anxiety that the subject has had during the past week. Each item of the BAI is scored on a scale of 0 (not at all) to 3 (severely), with higher scores indicative of higher anxiety severity. The original and the Italian versions showed good internal consistency (Cronbach’s *α* = 0.92 and 0.89, respectively) and adequate test–retest reliability. The current study showed good internal consistency (Cronbach’s *α* = 0.91; McDonald’s ω_h_ = 0.59; McDonald’s ω_t_ = 0.93; Flora, 2020).

The Beck Depression Inventory-II (BDI-II; Beck et al., 1996; Ghisi et al., 2006) is a 21-items self-report questionnaire, rated on a four-point Likert scale ranging from 0 to 3, used to assess the severity of depressive symptoms. Higher scores indicate higher depressive symptoms severity. The original and the Italian versions showed excellent internal consistency (Cronbach’s *α* = 0.93 and 0.87, respectively) and test–retest reliability (*r* = 0.93 and 0.76, respectively). In the current study, the questionnaire proved good internal consistency (Cronbach’s *α* = 0.88; McDonald’s ω_h_ = 0.57; McDonald’s ω_t_ = 0.90; Flora, 2020).

The Impact of Event Scale-Revised (IES-R; Weiss and Marmar, 1997; Pietrantonio et al., 2003) is a 22-item self-report measure that assesses subjective distress caused by traumatic events. Respondents are asked to identify a specific stressful life event and then indicate how distressed or bothered they were during the past 7 days by each “difficulty” listed. Items are rated on a 5-point scale ranging from 0 (“not at all”) to 4 (“extremely”). The IES-R yields a total score (ranging from 0 to 88), and subscale scores can also be calculated for the Intrusion, Avoidance, and Hyperarousal subscales. A score above 50 indicates a probable case of PTSD. Internal consistency in the current study was from 0.82 to 0.90, for Cronbach’s α; from 0.65 to 0.82 for McDonald’s ω_h_; from 0.88 to 0.95 for McDonald’s ω_t_ (Flora, 2020).

### Statistical analysis

Statistical analyzes were performed using the SAS statistical package (SAS Institute Inc., 2013), RStudio (RStudio Team, 2020), and R software (R Core Team, 2020). Clinical and demographic variables are described using the median and interquartile ranges for quantitative data, frequencies, and percentages for categorical data. The PSWQ and IUS-R scales were analyzed as continuous and dichotomous variables to assess their distribution within socio-demographics, clinical, and treatment variables and to define patient groups to be compared in terms of worry content, anxiety, depression, and distress caused by traumatic events, respectively.

Linear regression models were used to evaluate the impact of clinical characteristics on trait variables at diagnosis. Mixed-effects linear models were fitted for each measure to examine the change within and between groups of interest. A mixed-effects logistic model analyzed the presence of specific stressful life events reported over time and between patients’ characteristics. The mixed models included the time of questionnaire assessment, the group, and their interaction, as fixed effects, and an unstructured covariance structure for the random effects.

All statistical tests used a two-sided 1% significance level (2-sided) to reduce the possibility of false-positive statistical testing.

## Results

### Patient characteristics

The entire sample is composed of 150 patients. No statistically significant differences between patients who returned the questionnaires and those who did not regarding sociodemographic and clinical variables were observed (Supplementary Table S1). The baseline characteristics of patients are summarized in Table 1. Most of them had stage I-II invasive breast carcinoma (81.7%), and out of 120 patients with available data for surgery, 41 (34.2%) had a mastectomy.

**Table 1:** Demographics and clinical characteristics of patients at enrolment (*N* = 150).

		Total (*N* = 150)	
Age	Median (Q1, Q3)	52.4 (46.2, 61.9)	
	<=50	57	38.0%
	>50	93	62.0%
Marital status	Married/Living together	116	77.3%
	Unmarried/Widower/Divorced	34	22.7%
Education	High school/ University	96	64.0%
	Primary/Secondary school	54	36.0%
Job	Employed	82	54.7%
	Not employed/ Retired	68	45.3%
Living	Alone	20	13.3%
	With partner/family	130	86.7%
Stage	N-Miss	18	
	0	13	9.8%
	IA-IB	81	61.4%
	IIA-IIB	30	22.7%
	IIIA-IIIB-IIIC	7	5.3%
	IV	1	0.8%
Type of surgery	N-Miss	30	
	Conservative	79	65.8%
	Mastectomy	41	34.2%

### Trait measures

The Worry and IU trait constructs, measured by the PSWQ and the IUS-R scales, remained stable over time, and the baseline assessment did not differ among patients’ characteristics (Supplementary Tables S2, S3).

## Impact of intolerance of uncertainty and trait of worry on psychopathological symptoms

### Worry contents

**Figure 1:**
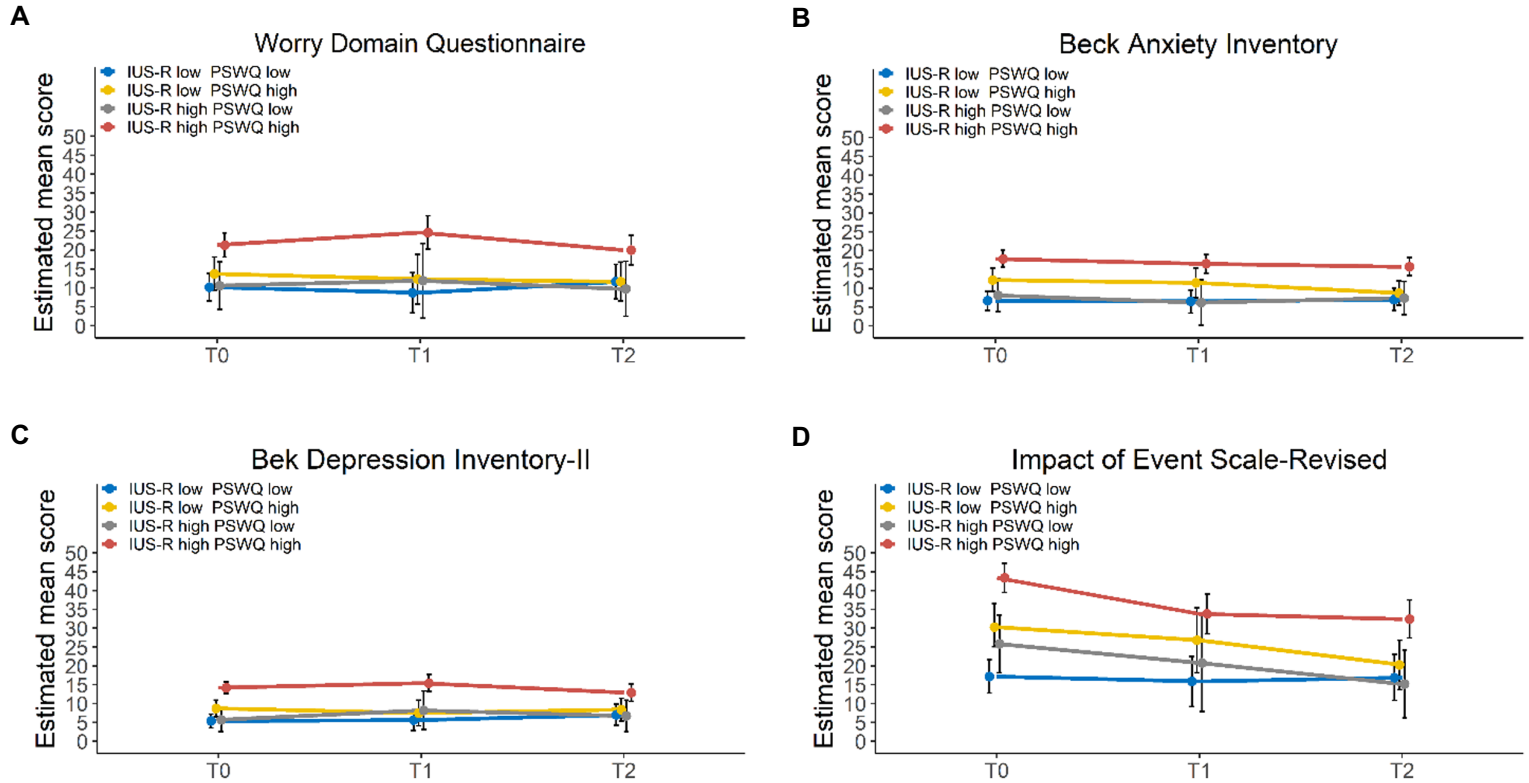
Trends over time of the WDQ **(A)**, BAI **(B)**, BDI-II **(C)**, and IES-R **(D)** scores of 4 groups divided based on the PSWQ and the IUS-R clinical cut-off (T0: *N* = 150; T1: *N* = 85; T2: *N* = 96). PSWQ = Penn State Worry Questionnaire; IUS-R = Intolerance of Uncertainty-Revised. T0: at diagnosis, T1: 3 months post-diagnosis, T2: 12 months post-diagnosis.

The WDQ total and subscales scores remained unchanged across the study period, except for the subscale “Lack of confidence” which increased during treatment for patients who have higher baseline IUS-R levels (at diagnosis: 4.6 points, 95% CI: 3.8 to 5.4; 3 months post-diagnosis: 5.5 points, 95% CI: 4.4 to 6.5), and decreased at follow-up (12 months post-diagnosis: 4.0 points, 95% CI: 3.0 to 5.0; *p* = 0.0017) and for patients reporting both a higher baseline IUS-R and PSWQ levels (at diagnosis: 5.1 points, 95% CI: 4.2 to 5.9; 3 months post-diagnosis: 6.1 points, 95% CI: 4.9 to 7.2; 12 months post-diagnosis: 4.5 points, 95% CI: 3.4 to 5.6; *p* = 0.0023; Figure 1A; Table 2; Supplementary Table S4).

**Table 2:** Mean changes over time and across clinical characteristics of the worry content, anxiety, depression, and post-traumatic stress symptom scales (T0: *N* = 150; T1: *N* = 85; T2: *N* = 96).

	Overall	T0	*N*	T1	*N*	T2	*N*	Value of *p* within
Worry Domain Questionnaire		15.3 (13.2–17.4)	150	16.2(13.4–19.3)	85	14.7 (12.2–17.1)	96	0.4065
Baseline PSWQ low	10.2 (6.7–13.6)	10.3 (7.1–13.5)	60	9.2 (4.4–14.0)	31	11.0 (7.0–15.0)	37	0.6361
Baseline PSWQ high	18.8 (16–21.6)	18.6 (16.0–21.2)	90	20.7 (17.0–24.5)	54	17.1 (14.0–20.2)	59	0.0481
Value of *p* between	***0.0002***	***0.0001***		***0.0003***		0.0171		
Baseline IUS-R low	11.1 (8–14.2)	11.7 (8.8–14.5)	77	10.1 (5.9–14.3)	39	11.5 (8.0–15.0)	47	0.5906
Baseline IUS-R high	19.7 (16.6–22.8)	19.1 (16.2–22.0)	73	22.2 (18.1–26.2)	46	17.9 (14.4–21.3)	49	0.0234
Value of *p* between	***0.0001***	***0.0005***		***<0.0001***		0.0114		
Baseline PSWQ & IUS-R low	10.2 (6.3–14.1)	10.2 (6.6–13.8)	45	8.7 (3.4–14.0)	25	11.7 (7.1–16.2)	25	0.3727
Baseline PSWQ high & baseline IUS-R low	12.6 (7.9–17.2)	13.7 (9.4–18.1)	32	12.3 (5.7–18.9)	14	11.6 (6.5–16.8)	22	0.6033
Baseline PSWQ low & baseline IUS-R high	10.7 (4.0–17.5)	10.6 (4.3–16.9)	15	11.9 (2.0–21.7)	6	9.7 (2.5–17.0)	12	0.8563
Baseline PSWQ & IUS-R high	22.0 (18.6–25.3)	21.3 (18.1–24.5)	58	24.6 (20.2–29.0)	40	20.0 (16.1–23.8)	37	0.0269
Value of *p* between	***<0.0001***	***<0.0001***		***<0.0001***		***0.0078***		
Beck anxiety inventory		12.3 (10.7–13.9)	150	11.6 (9.8–13.4)	85	10.9 (9.2–12.6)	96	0.3210
Baseline PSWQ low	6.9 (5–8.7)	7.0 (4.8–9.3)	60	6.4 (3.7–9.2)	31	7.2 (4.6–9.7)	37	0.8828
Baseline PSWQ high	14.7 (13.2–16.1)	15.9 (14.0–17.7)	90	14.8 (12.8–16.9)	54	13.3 (11.2–15.3)	59	0.0965
Value of *p* between	***<0.0001***	***<0.0001***		***<0.0001***		***0.0003***		
Baseline IUS-R low	8.4 (6.6–10.1)	9.0 (6.9–11.1)	77	8.4 (5.8–10.9)	39	7.7 (5.4–10.0)	47	0.6342
Baseline IUS-R high	14.8 (13.2–16.5)	15.8 (13.7–18.0)	73	14.7 (12.4–17.1)	46	14.0 (11.7–16.2)	49	0.3513
Value of *p* between	***<0.0001***	***<0.0001***		***0.0004***		***0.0002***		
Baseline PSWQ & IUS-R low	6.7 (4.7–8.8)	6.7 (4.1–9.2)	45	6.5 (3.4–9.5)	25	7.0 (4.0–10.0)	25	0.9518
Baseline PSWQ high & baseline IUS-R low	10.8 (8.4–13.2)	12.2 (9.2–15.3)	32	11.4 (7.4–15.4)	14	8.7 (5.5–11.9)	22	0.1902
Baseline PSWQ low & baseline IUS-R high	7.2 (3.7–10.7)	8.1 (3.7–12.5)	15	6.2 (0.2–12.2)	6	7.4 (2.9–11.8)	12	0.8532
Baseline PSWQ & IUS-R high	16.7 (15–18.4)	17.8 (15.6–20.1)	58	16.5 (14.0–18.9)	40	15.7 (13.3–18.2)	37	0.3504
Value of *p* between	***<0.0001***	***<0.0001***		***<0.0001***		***<0.0001***		
Beck depression inventory-II		9.5 (8.3–10.7)	150	10.2 (8.6–11.8)	85	9.6 (8.1–11.0)	96	0.5102
Baseline PSWQ low	6.2 (4.4–7.9)	5.5 (3.8–7.1)	60	6.1 (3.6–8.6)	31	6.9 (4.6–9.2)	37	0.4668
Baseline PSWQ high	12.1 (10.7–13.6)	12.2 (10.9–13.6)	90	12.9 (10.9–14.8)	54	11.3 (9.5–13.2)	59	0.2716
Value of *p* between	***<0.0001***	***<0.0001***		***<0.0001***		***0.0037***		
Baseline IUS-R low	6.9 (5.3–8.5)	6.8 (5.2–8.3)	77	6.4 (4.2–8.6)	39	7.6 (5.5–9.7)	47	0.5190
Baseline IUS-R high	12.6 (11.1–14.2)	12.4 (10.9–14.0)	73	13.9 (11.8–16.0)	46	11.5 (9.5–13.6)	49	0.0463
Value of *p* between	***<0.0001***	***<0.0001***		***<0.0001***		***0.0085***		
Baseline PSWQ & IUS-R low	6 (4–8)	5.4 (3.5–7.2)	45	5.6 (2.9–8.3)	25	7.0 (4.3–9.8)	25	0.4744
Baseline PSWQ high & baseline IUS-R low	8.2 (5.9–10.5)	8.7 (6.5–10.9)	32	7.5 (4.1–10.9)	14	8.4 (5.4–11.4)	22	0.6974
Baseline PSWQ low & baseline IUS-R high	6.9 (3.5–10.3)	5.7 (2.6–8.9)	15	8.2 (3.1–13.3)	6	6.7 (2.6–10.9)	12	0.5217
Baseline PSWQ & IUS-R high	14.2 (12.5–15.8)	14.2 (12.6–15.8)	58	15.4 (13.2–17.7)	40	12.9 (10.6–15.2)	37	0.0720
Value of *p* between	***<0.0001***	***<0.0001***		***<0.0001***		***0.0042***		
Impact of event scale-revised		31.0 (28.0–34.0)	150	25.5 (21.8–29.2)	85	23.3 (20.0–26.7)	96	<0.0001
Baseline PSWQ low	17.5 (13.7–21.3)	19.4 (15.3–23.4)	60	17.0 (11.1–22.8)	31	16.2 (11.0–21.4)	37	0.4721
Baseline PSWQ high	32.6 (29.6–35.6)	38.7 (35.4–42.0)	90	31.1 (26.6–35.6)	54	28.0 (23.9–32.1)	59	<0.0001
Value of *p* between	***<0.0001***	***<0.0001***		***0.0002***		***0.0006***		
Baseline IUS-R low	20.3 (16.9–23.8)	22.6 (19.0–26.3)	77	20.2 (14.9–25.5)	39	18.2 (13.5–22.8)	47	0.1871
Baseline IUS-R high	33.2 (29.7–36.6)	39.8 (36.0–43.5)	73	31.1 (26.1–36.1)	46	28.6 (24.0–33.2)	49	<0.0001
Value of *p* between	***<0.0001***	***<0.0001***		***0.0037***		***0.0020***		
Baseline PSWQ & IUS-R l	16.6 (12.4–20.9)	17.2 (12.8–21.6)	45	15.9 (9.2–22.5)	25	16.9 (10.8–23.0)	25	0.9139
Baseline PSWQ high & baseline IUS-R low	25.8 (20.7–30.8)	30.3 (25.1–36.5)	32	26.8 (18.2–35.4)	14	20.3 (13.7–26.8)	22	0.0277
Baseline PSWQ low & baseline IUS-R high	20.6 (13.3–28)	25.9 (18.2–33.5)	15	20.8 (7.9–33.8)	6	15.2 (6.2–24.2)	12	0.1163
Baseline PSWQ & IUS-R high	36.5 (33–40.1)	43.4 (39.5–47.2)	58	33.8 (28.5–39.1)	40	32.4 (27.4–37.5)	37	***<0.0001***
Value of *p* between	***<0.0001***	***<0.0001***		***0.0007***		***0.0002***		

The bold values are mean statistically significant *p* < 0.01. PSWQ = Penn State Worry Questionnaire; IUS-R = Intolerance of Uncertainty-Revised. T0: at diagnosis, T1: three months post-diagnosis, T2: 12 months post-diagnosis. Values are mean estimates with their 95% confidence interval.

Overall, patients reporting elevated baseline PSWQ or/and IUS-R levels had a statistically significantly worse WDQ score at T0 that remained impaired throughout the study (Table 2). Similar patterns were observed for WDQ “Lack of confidence,” “Aimless future,” and “Work” subscales. This impairment was observed for the WDQ “Financial” subscale only for patients with pathological PSWQ (Supplementary Table S4).

### Anxiety and depressive symptoms

The BAI and the BDI-II scores (Figures 1B,C; Table 2) did not significantly change over time. Similarly to what was observed for the WDQ scale, high PSWQ and/or IUS-R scores were significantly associated with a detrimental impact on anxiety and depressive symptoms.

### Post-traumatic stress disorder symptoms

The IES-R scale and all subscales displayed a statistically significant reduction across the study period. This improvement was observed in particular for patients reporting high PSWQ and/or IUS-R scores at diagnosis (Figure 1D; Table 2; Supplementary Table S5). Moreover, high PSWQ and/or IUS-R levels significantly worsened distress caused by traumatic events (Table 2).

Considering the specific stressful life events identified in the IES–R, disease was reported at diagnosis by 36.7% of patients. This percentage decreased to 20% 12 months post-diagnosis, although not statistically significant (Table 3).

**Table 3:** Frequency of events reported over time: overall and according to patient characteristics (T0: *N* = 150; T1: *N* = 85; T2: *N* = 96).

	Overall	T0	*N*	T1		T2		Value of *p* within
IES events								
Disease	102 (30.8%)	55 (36.7%)	150	27 (31.8%)	85	20 (20.7%)	96	0.0244
Age < =50 years	34 (28.1%)	21 (36.8%)	57	6 (22.2%)	27	7 (18.9%)	37	0.0766
Age > 50 years	68 (32.4%)	34 (36.6%)	93	21 (36.2%)	58	13 (22%)	59	0.1297
Value of *p* between	0.3594	0.9731		0.1604		0.6822		
Conservative surgery	54 (30.7%)	29 (36.7%)	79	17 (35.4%)	48	8 (16.3%)	49	0.0338
Mastectomy	28 (30.4%)	14 (34.1%)	41	6 (25.0%)	24	8 (29.6%)	27	0.6288
Value of *p* between	0.9961	0.7900		0.2664		0.2133		
Married/Living together	79 (30.7%)	44 (37.9%)	116	20 (31.2%)	64	15 (19.5%)	77	0.0284
Unmarried/Widower/Divorced	23 (31.1%)	11 (32.3%)	34	7 (33.3%)	21	5 (26.3%)	19	0.7169
Value of *p* between	0.8712	0.5665		0.7518		0.6994		
Primary/Secondary school	31 (27.4%)	17 (31.5%)	54	11 (36.7%)	30	3 (10.3%)	29	0.0974
High school/University	71 (32.6%)	38 (39.6%)	96	16 (29.1%)	55	17 (25.4%)	67	0.1270
Value of *p* between	0.3091	0.3406		0.5553		0.1603		
Employed	60 (32.8%)	34 (41.5%)	82	15 (31.2%)	48	11 (20.7%)	53	0.0484
Not employed/Retired	42 (28.4%)	21 (30.9%)	68	12 (32.4%)	37	9 (20.9%)	43	0.4015
Value of *p* between	0.6241	0.1977		0.9325		0.9686		
Living alone	13 (29.5%)	5 (25.0%)	20	5 (41.7%)	12	3 (25.0%)	12	0.5402
With partner/family	89 (31%)	50 (38.5%)	130	22 (30.1%)	73	17 (20.2%)	84	0.0198
Value of *p* between	0.9054	0.2676		0.3950		0.7956		
Baseline PSWQ low	30 (23.4%)	15 (25.0%)	60	8 (25.8%)	31	7 (18.9%)	37	0.4964
Baseline PSWQ high	72 (35.5%)	40 (44.4%)	90	19 (35.2%)	54	13 (22.0%)	59	0.0229
Value of *p* between	0.1476	0.0218		0.6231		0.6331		
Baseline IUS-R low	40 (24.5%)	24 (31.2%)	77	9 (23.1%)	39	7 (14.9%)	47	0.1138
Baseline IUS-R high	62 (36.9%)	31 (42.5%)	73	18 (39.1%)	46	13 (26.5%)	49	0.1815
Value of *p* between	0.0397	0.1676		0.1595		0.156941		
Baseline PSWQ & IUS-R low	18 (18.9%)	11 (24.4%)	45	4 (16.0%)	25	3 (12.0%)	25	0.4242
Baseline PSWQ high & baseline IUS-R low	22 (32.3%)	13 (40.6%)	32	5 (35.7%)	14	4 (18.2%)	22	0.2043
Baseline PSWQ low & baseline IUS-R high	12 (36.4%)	4 (26.7%)	15	4 (66.7%)	6	4 (33.3%)	12	0.2430
Baseline PSWQ & IUS-R high	50 (37.0%)	27 (46.5%)	58	15 (35.0%)	40	9 (24.3%)	37	0.0703
Value of *p* between	0.1193	0.1533		0.2068		0.4221		

PSWQ = Penn State Worry Questionnaire; IUS-R = Intolerance of Uncertainty-Revised. T0: at diagnosis, T1: three months post-diagnosis, T2: 12 months post-diagnosis.

## Discussion

The current study investigated the trait of worry, intolerance of uncertainty, depression, anxiety, and post-traumatic symptoms related to breast cancer and how they change from diagnosis through treatment and follow-up. Considering that worry and IU are dispositional traits, we also aimed to understand whether breast cancer patients with a difficulty in managing uncertainty and worry traits showed high levels of worry content, depression, anxiety, and post-traumatic stress symptoms over time.

First, we demonstrated a longitudinal stability of the trait constructs measured by the PSWQ and the IUS-R scales, in line with studies that showed how IU and trait of worry seems to be stable over time and across situation (e.g., Dugas et al., 2004; Mahoney and McEvoy, 2012; Brintzinger et al., 2021; Lauriola et al., 2023). Coherently with literature (e.g., Carleton et al., 2012), we evidenced that the trait of worry and intolerance of uncertainty were related to high levels of worry contents, anxiety, and depressive symptoms, underlying the connection between IU and trait of worry with other psychopathological symptoms. Therefore, IU and the trait of worry should be considered two relevant aspects that could help to discriminate between patients who maintain high depression, anxiety, and post-traumatic disorders scores. These data align with the description of the IU and trait of worry as trans-diagnostic constructs identified as factors that play a central role in developing and maintaining different psychopathology (such as obsessive–compulsive disorder, generalized anxiety disorder, and depression), in line with other studies that focused their investigation on sample with and without the presence of a cancer diagnosis (e.g., Eisenberg et al., 2015; Carleton, 2016a,b; Cessna Palas et al., 2021). If our outcomes are confirmed, screening and intervention in preventing dysfunctional responses to stressors, as are the diagnostic and treatment processes related to breast cancer, could be considered valid procedures that improve the patients’ quality of life.

Our results are in line with what emerged in the literature related to the communication of the cancer phase (e.g., Gieseler et al., 2018), also highlighting how this moment is a crucial, sensitive, and challenging situation for people, independently of the socio-demographic variables. Indeed, cancer events described as traumatic experiences are particularly salient during the period close to the communication of the diagnosis than during the treatment period. The diagnosis communication could be perceived as a discrepancy between what is happening and the ability to cope with the event (Gieseler et al., 2018). The fact that the cancer event is less referred to as a traumatic episode in the subsequent assessment phases could be interpreted as a physiological process of habituation and elaboration, concurrently with the planning of treatment. Although with a different method and timing, a similar trend has been observed by Chan and colleagues Chan et al. (2018), who have analyzed PTSD features over 4 years, showing a general decrease in symptomatology. These data confirm the need to consider cancer as a traumatic event and to evaluate, in a systematic way, PTSD symptoms following a cancer diagnosis. Also, some socio-demographic features must be considered as protective factors of psychopathological manifestation related to cancer; in line with what has been highlighted by two studies (Cordova et al., 2017; Van Oers, 2019), social support, living together, and socio-economic status could play a role as predisposition factors in the development of PTSD. Although it is important to consider that no differences between groups have been shown for these variables, socio-demographic features, including a support network related to the person, have been considered influential elements of the quality of life, the general well-being, and the ability to manage cancer. Future studies could also considering to investigate the coping strategies and Posttraumatic Growth as possible risk and protective factors related to psychological symptoms. Moreover, it seems that intolerance of uncertainty and worry as a trait influenced patients’ mental health condition all the time, independently of the assessment phase and other features. For this reason, our outcome focus on the importance to consider IU and trait of worry as a potential therapeutic target (e.g., Brown et al., 2017), as well as the prominence in assessing and managing IU and trait of worry since the diagnosis phase to promote a better mental health condition of patients with a breast cancer diagnosis.

### Study limitations

A limitation of this study is the presence of questionnaires that need to be included. As in other studies with a longitudinal design, missing data increased over time. However, since 84% of patients completed the questionnaires at least in two assessment phases, the potential biasing effect of missing data may have been less strongly affected by the results of the comparisons over time and the overall mean between groups.

### Clinical implications

The presence of clinically significant worry as a trait and IU features at the beginning of the breast cancer diagnostic procedure should be carefully considered in the intervention planning by the clinician. The systematic assessment of worry and IU could be helpful in managing the psychological problems that during treatment and follow-up stages tend to remain high and seem precisely influenced by traits such as intolerance for uncertainty and the trait of worry.

## Conclusion

Globally, our results indicate that processing traits variables, such as worry and intolerance of uncertainty, can have a significant impact on the global psychological condition of patients. An early assessment of the components of worry as a trait and intolerance of uncertainty could be important in identifying those people with a higher psychopathological risk (e.g., Brown et al., 2017). In addition, monitoring and support throughout the disease would be desirable if further studies confirmed these data. Overall, outcomes show the importance of promoting specific psychological rehabilitation protocols for breast cancer patients’ global well-being (Ramírez-Vélez et al., 2021).

## Data availability statement

The raw data supporting the conclusions of this article will be made available by the authors, without undue reservation.

## Ethics statement

The current study was conducted following the Declaration of Helsinki and the protocol n. 642 identified by IOV-BC-1-2015 IT&W and approved by the IOV Ethical Committee on 12/15/2015. The patients/participants provided their written informed consent to participate in this study.

## Author contributions

SP, CN, and MS: conceptualization of the protocol. MS, SP, and CN: methodology. CC and SP: investigation. PDB and GLDS: curation and formal analysis. SP and CC: writing and original draft preparation. CN, PDB, and MS: supervised. All authors contributed to the manuscript editing and revision, and they have read and agreed to the published version of the manuscript.

## Funding

This research was funded by Italian Ministry of Health Ricerca Corrente.

## Conflict of interest

The authors declare that the research was conducted in the absence of any commercial or financial relationships that could be construed as a potential conflict of interest.

## Publisher’s note

All claims expressed in this article are solely those of the authors and do not necessarily represent those of their affiliated organizations, or those of the publisher, the editors and the reviewers. Any product that may be evaluated in this article, or claim that may be made by its manufacturer, is not guaranteed or endorsed by the publisher.
