# Supplementary material for: The predictive role of intolerance of uncertainty and trait of worry in breast cancer patients: A prospective, observational, single-center clinical study
Source: Front Psychol. 2023 Apr 17;14:1092060. doi: 10.3389/fpsyg.2023.1092060 (PMC10149753; doi:10.3389/fpsyg.2023.1092060)
Supplement: Supplementary file 1 [file Data_Sheet_1.pdf]

Supplementary materials:

Table S1. Demographics and clinical characteristics between patients with and without available questionnaires

		Missing at T1 (N=65)	Available at T1 (N=85)	*p value*	Missing at T2 (N=54)	Available at T2 (N=96)	*p value*
**age**	Median (Q1, Q3)	50.6 (44.9, 59.6)	52.8 (47.0, 64.4)	*0.0810*	53.2 (46.4, 61.1)	51.9 (46.0, 62.4)	*0.7840*
**Marital status**	Married	52 (80.0%)	64 (75.3%)	*0.4950*	39 (72.2%)	77 (80.2%)	*0.2620*
	Unmarried	13 (20.0%)	21 (24.7%)		15 (27.8%)	19 (19.8%)	
**Education**	High	41 (63.1%)	55 (64.7%)	*0.8370*	29 (53.7%)	67 (69.8%)	*0.0490*
	Primary	24 (36.9%)	30 (35.3%)		25 (46.3%)	29 (30.2%)	
**Job**	Employed	34 (52.3%)	48 (56.5%)	*0.6120*	29 (53.7%)	53 (55.2%)	*0.8590*
	Not Employed	31 (47.7%)	37 (43.5%)		25 (46.3%)	43 (44.8%)	
**Living**	Alone	8 (12.3%)	12 (14.1%)	*0.7470*	8 (14.8%)	12 (12.5%)	*0.6890*
	With family	57 (87.7%)	73 (85.9%)		46 (85.2%)	84 (87.5%)	
**Stage**	NMiss	12	6		8	10	
	0	5 (9.4%)	8 (10.1%)	*0.1040*	6 (13.0%)	7 (8.1%)	*0.6660*
	IAIB	36 (67.9%)	45 (57.0%)		29 (63.0%)	52 (60.5%)	
	IIAIIB	7 (13.2%)	23 (29.1%)		8 (17.4%)	22 (25.6%)	
	IIIAIIIBIIIC	5 (9.4%)	2 (2.5%)		3 (6.5%)	4 (4.7%)	
	IV	0 (0.0%)	1 (1.3%)		0 (0.0%)	1 (1.2%)	
**Type of surgery**	NMiss	17	13		10	20	
	Conservative	31 (64.6%)	48 (66.7%)	*0.8140*	30 (68.2%)	49 (64.5%)	*0.6800*
	Mastectomy	17 (35.4%)	24 (33.3%)		14 (31.8%)	27 (35.5%)	
**PSWQ**	Median (Q1, Q3)	41.0 (34.0, 53.0)	45.0 (36.0, 54.0)	*0.3840*	41.5 (35.2, 52.0)	43.5 (35.0, 55.0)	*0.4030*
**IUS-R**	Median (Q1, Q3)	25.0 (21.0, 31.0)	28.0 (20.0, 34.0)	*0.1690*	25.0 (20.0, 30.7)	27.5 (21.0, 33.0)	*0.2870*

PSWQ= Penn State Worry Questionnaire; IUS-R= Intolerance of Uncertainty-Revised.

Table S2. Distribution of trait measures (T0: N=150; T1: N=85; T2: N=96).

Variable	Time	N	Mean	Std Dev	Median	Lower Quartile	Upper Quartile
**PSWQ**	T0	150	45.3	11.8	43	36	54
	T1	85	45	12	43	36	52
	T2	96	43	12.1	42	35	51
**IUS-R**	T0	150	27.6	9.6	27	21	32
	T1	85	27.1	10.4	23	19	36
	T2	96	25.3	9.7	23	19	28

PSWQ= Penn State Worry Questionnaire; IUS-R= Intolerance of Uncertainty-Revised.

Table S3. Baseline PSWQ and IUS-R scales across patient characteristics (N=150)

Variable			PSWQ		IUS-R	
		N	Estimate (95%CI)	p-value	Estimate (95%CI)	*p-value*
**Age**	<=50 years	57	44.4 (41.3-47.5)	0.4661	25.3 (22.9-27.8)	*0.0214*
	> 50 years	93	45.9 (43.4-48.3)		29.0 (27.1-31.0)	
**Type of surgery**	Conservative	79	46.1 (43.5-48.7)	0.1061	29.1 (26.9-31.2)	*0.0955*
	Mastectomy	41	42.4 (38.8-46.1)		25.9 (22.9-28.9)	
**Marital status**	Married/Living together	116	45.9 (43.8-48.1)	0.2211	27.8 (26.0-29.6)	*0.6542*
	Unmarried/Widower/Divorced	34	43.1 (39.1-47.1)		27.0 (23.7-30.2)	
**Education**	Primary/Secondary school	54	44.3 (41.1-47.5)	0.4347	28.5 (25.9-31.1)	*0.4100*
	High school/ University	96	45.9 (43.5-48.3)		27.1 (25.2-29.1)	
**Job**	Employed	82	45.6 (43.0-48.2)	0.7631	27.3 (25.2-29.4)	*0.6593*
	Not employed/ Retired	68	45.0 (421-47.8)		28.0 (25.7-30.3)	
**Living**	Alone	20	42.1 (36.9-47.4)	0.2010	28.8 (24.6-33.1)	*0.5386*
	With partner/family	130	45.8 (43.7-47.8)		27.4 (25.8-29.1)	

PSWQ= Penn State Worry Questionnaire; IUS-R= Intolerance of Uncertainty-Revised.

Table S4. The WDQ subscales over time across high and low baseline PSWQ and IUS-R levels (T0: N=150; T1: N=85; T2: N=96).

	**Overall**	**T0**	**N**	**T1**	**N**	**T2**	**N**	***p-value within***
**WDQ Relationships**		2 (1.6-2.4)	150	2.2 (1.6-2.8)	85	2.2 (1.7-2.8)	96	*0.4861*
baseline PSWQ low	1.4 (0.7-2.1)	1.7 (1-2.3)	60	1.2 (0.2-2.2)	31	1.4 (0.6-2.2)	37	*0.4415*
baseline PSWQ high	2.6 (2.1-3.2)	2.2 (1.7-2.8)	90	2.9 (2.1-3.6)	54	2.8 (2.1-3.4)	59	*0.0478*
p-value between	***0.0062***	*0.1764*		***0.0077***		***0.0099***		
baseline IUS-R low	1.5 (0.9-2.1)	1.6 (1-2.2)	77	1.2 (0.3-2)	39	1.7 (1-2.5)	47	*0.3537*
baseline IUS-R high	2.8 (2.2-3.4)	2.5 (1.9-3)	73	3.2 (2.4-4)	46	2.7 (2-3.5)	49	*0.0535*
p-value between	***0.0023***	*0.0405*		***0.0005***		*0.0563*		
baseline PSWQ & IUS-R low	1.5 (0.7-2.2)	1.7 (0.9-2.4)	45	1.1 (0.1-2.2)	25	1.5 (0.6-2.5)	25	*0.4503*
baseline PSWQ high & baseline IUS-R low	1.5 (0.6-2.5)	1.5 (0.6-2.4)	32	1.2 (-0.1-2.6)	14	1.9 (0.8-3)	22	*0.5177*
baseline PSWQ low & baseline IUS-R high	1.4 (0.1-2.7)	1.6 (0.3-2.9)	15	1.6 (-0.4-3.6)	6	1.1 (-0.5-2.6)	12	*0.6919*
baseline PSWQ & IUS-R high	3.2 (2.5-3.8)	2.7 (2-3.3)	58	3.6 (2.7-4.5)	40	3.2 (2.4-4.1)	37	*0.0251*
p-value between	***0.0020***	*0.0919*		***0.0017***		*0.0173*		
**WDQ lack of confidence**		3.5 (3-4.1)	150	3.7 (2.9-4.5)	85	3.3 (2.6-4)	96	*0.3174*
baseline PSWQ low	2.2 (1.2-3.1)	2.3 (1.5-3.2)	60	1.9 (0.6-3.2)	31	2.3 (1.2-3.4)	37	*0.6069*
baseline PSWQ high	4.4 (3.6-5.1)	4.3 (3.6-5.1)	90	4.9 (3.9-5.9)	54	3.9 (3-4.7)	59	*0.0341*
p-value between	***0.0007***	***0.0007***		***0.0004***		*0.0358*		
baseline IUS-R low	2.3 (1.5-3.1)	2.6 (1.8-3.3)	77	1.9 (0.8-3)	39	2.5 (1.5-3.5)	47	*0.2204*
baseline IUS-R high	4.7 (3.8-5.5)	4.6 (3.8-5.4)	73	5.5 (4.4-6.5)	46	4.0 (3.0-5.0)	49	***0.0017***
p-value between	***0.0001***	***0.0005***		***<0.0001***		*0.0288*		
baseline PSWQ & IUS-R low	2.1 (1.1-3.2)	2.2 (1.2-3.2)	45	1.7 (0.3-3.1)	25	2.5 (1.2-3.8)	25	*0.3345*
baseline PSWQ high & baseline IUS-R low	2.6 (1.3-3.8)	3 (1.8-4.2)	32	2.1 (0.4-3.9)	14	2.5 (1-4)	22	*0.4226*
baseline PSWQ low & baseline IUS-R high	2.5 (0.7-4.4)	2.7 (0.9-4.4)	15	2.8 (0.3-5.4)	6	2.1 (0-4.2)	12	*0.6912*
baseline PSWQ & IUS-R high	5.2 (4.3-6.1)	5.1 (4.2-5.9)	58	6.1 (4.9-7.2)	40	4.5 (3.4-5.6)	37	***0.0023***
p-value between	***0.0001***	***0.0003***		***<0.0001***		*0.0373*		
**WDQ Aimless future**		3.2 (2.7-3.7)	150	3.4 (2.7-4.1)	85	2.9 (2.3-3.5)	96	*0.2426*
baseline PSWQ low	2 (1.2-2.8)	2.1 (1.3-2.9)	60	2 (0.9-3.1)	31	2 (1.1-3)	37	*0.9558*
baseline PSWQ high	3.9 (3.2-4.6)	3.9 (3.3-4.6)	90	4.3 (3.4-5.1)	54	3.5 (2.8-4.3)	59	*0.0877*
p-value between	***0.0006***	***0.0004***		***0.0014***		*0.0154*		
baseline IUS-R low	2.3 (1.6-3.1)	2.6 (1.9-3.3)	77	2.1 (1.2-3.1)	39	2.3 (1.4-3.1)	47	*0.4377*
baseline IUS-R high	4 (3.2-4.7)	3.8 (3.1-4.5)	73	4.5 (3.6-5.4)	46	3.6 (2.7-4.4)	49	*0.0219*
p-value between	***0.0024***	0.019		***0.0005***		*0.0371*		
baseline PSWQ & IUS-R low	2.1 (1.1-3)	2.1 (1.2-3)	45	1.9 (0.7-3.1)	25	2.2 (1.1-3.4)	25	*0.7388*
baseline PSWQ high & baseline IUS-R low	2.7 (1.6-3.8)	3.3 (2.2-4.3)	32	2.5 (1-4)	14	2.4 (1.1-3.7)	22	*0.2507*
baseline PSWQ low & baseline IUS-R high	2 (0.4-3.6)	1.9 (0.4-3.5)	15	2.5 (0.2-4.7)	6	1.6 (-0.2-3.3)	12	*0.5673*
baseline PSWQ & IUS-R high	4.5 (3.7-5.3)	4.3 (3.5-5.1)	58	5 (4-6)	40	4.1 (3.2-5)	37	*0.0452*
p-value between	***0.0006***	***0.0019***		***0.0006***		*0.016*		
**WDQ Work**		3.9 (3.4-4.5)	150	4.1 (3.3-4.9)	85	3.6 (3-4.2)	96	*0.3806*
baseline PSWQ low	2.7 (1.9-3.5)	2.6 (1.8-3.3)	60	2.5 (1.3-3.7)	31	3.1 (2.2-4.1)	37	*0.3985*
baseline PSWQ high	4.6 (4-5.3)	4.8 (4.2-5.5)	90	5.1 (4.2-6)	54	3.9 (3.2-4.7)	59	*0.0159*
p-value between	***0.0003***	***<0.0001***		***0.001***		*0.2088*		
baseline IUS-R low	2.9 (2.2-3.6)	2.8 (2.1-3.5)	77	2.9 (1.8-4)	39	2.9 (2-3.8)	47	*0.9624*
baseline IUS-R high	4.9 (4.2-5.6)	5.1 (4.4-5.8)	73	5.3 (4.3-6.3)	46	4.4 (3.5-5.2)	49	*0.1062*
p-value between	***<0.0001***	***<0.0001***		***0.0018***		*0.0182*		

baseline PSWQ & IUS-R low	2.8 (1.9-3.7)	2.6 (1.7-3.5)	45	2.5 (1.2-3.9)	25	3.3 (2.1-4.4)	25	*0.4315*
baseline PSWQ high & baseline IUS-R low	3.1 (2-4.1)	3.1 (2.1-4.1)	32	3.6 (1.8-5.3)	14	2.6 (1.3-3.8)	22	*0.4339*
baseline PSWQ low & baseline IUS-R high	2.7 (1.1-4.2)	2.5 (0.9-4)	15	2.6 (0-5.2)	6	2.9 (1.2-4.7)	12	*0.8772*
baseline PSWQ & IUS-R high	5.5 (4.7-6.2)	5.8 (5-6.6)	58	5.9 (4.8-7)	40	4.7 (3.8-5.7)	37	*0.0435*
p-value between	***<0.0001***	***<0.0001***		***0.0009***		*0.0336*		
**WDQ Financial**		2.3 (1.8-2.8)	150	2.4 (1.8-3)	85	2.4 (1.9-3)	96	*0.9001*
baseline PSWQ low	1.6 (0.8-2.3)	1.5 (0.8-2.3)	60	1.3 (0.3-2.2)	31	1.9 (1-2.8)	37	*0.3603*
baseline PSWQ high	2.9 (2.3-3.5)	2.8 (2.2-3.5)	90	3.1 (2.3-3.8)	54	2.7 (2-3.5)	59	*0.6265*
p-value between	***0.0075***	*0.012*		***0.0038***		*0.1453*		
baseline IUS-R low	1.9 (1.2-2.6)	2 (1.3-2.7)	77	1.7 (0.8-2.5)	39	2 (1.2-2.8)	47	*0.5834*
baseline IUS-R high	2.8 (2.2-3.5)	2.7 (1.9-3.4)	73	3.1 (2.2-3.9)	46	2.8 (2-3.6)	49	*0.5029*
p-value between	*0.0490*	*0.1898*		*0.0195*		*0.1756*		
baseline PSWQ & IUS-R l	1.5 (0.7-2.4)	1.4 (0.5-2.4)	45	1.2 (0.1-2.3)	25	1.9 (0.9-3)	25	*0.324*
baseline PSWQ high & baseline IUS-R low	2.4 (1.4-3.5)	2.8 (1.7-3.8)	32	2.4 (1-3.7)	14	2.2 (1-3.4)	22	*0.5164*
baseline PSWQ low & baseline IUS-R high	1.8 (0.3-3.3)	1.8 (0.2-3.4)	15	1.6 (-0.4-3.7)	6	1.9 (0.2-3.5)	12	*0.9624*
baseline PSWQ & IUS-R high	3.1 (2.3-3.8)	2.9 (2.1-3.7)	58	3.3 (2.5-4.2)	40	3 (2.1-3.9)	37	*0.4514*
p-value between	*0.0487*	*0.0917*		*0.021*		*0.3739*		

Table S5. The IES-R subscales across high and low baseline PSWQ and IUS-R levels (T0: N=150; T1: N=85; T2: N=96).

	**Overall**	**T0**	**N**	**T1**	**N**	**T2**	**N**	***p-value within***
**IES intrusion**		11.9 (10.6-13.1)	150	9.9 (8.4-11.4)	85	9.6 (8.2-11.1)	96	***0.0027***
baseline PSWQ low	6.9 (5.3-8.4)	7.5 (5.8-9.2)	60	6.6 (4.2-9)	31	6.5 (4.3-8.7)	37	*0.6182*
baseline PSWQ high	12.8 (11.6-14.1)	14.8 (13.4-16.2)	90	12.1 (10.2-13.9)	54	11.7 (9.9-13.4)	59	***0.001***
p-value between	***<0.0001***	***<.0001***		***0.0005***		***0.0004***		
baseline IUS-R low	8 (6.6-9.4)	8.5 (7-10)	77	8 (5.8-10.2)	39	7.5 (5.5-9.5)	47	*0.6058*
baseline IUS-R high	13.1 (11.6-14.5)	15.4 (13.8-16.9)	73	11.9 (9.9-14)	46	11.8 (9.9-13.8)	49	***0.0002***
p-value between	***<0.0001***	***<.0001***		***0.0102***		***0.0027***		
baseline PSWQ & IUS-R low	6.4 (4.6-8.2)	6.6 (4.7-8.4)	45	6.2 (3.5-9)	25	6.4 (3.7-9)	25	*0.9683*
baseline PSWQ high & baseline IUS-R low	10.3 (8.2-12.4)	11.3 (9.1-13.5)	32	10.6 (7-14.1)	14	9 (6.2-11.9)	22	*0.3761*
baseline PSWQ low & baseline IUS-R high	8.2 (5.2-11.3)	10.3 (7.1-13.5)	15	7.5 (2.1-12.9)	6	6.9 (3-10.8)	12	*0.261*
baseline PSWQ & IUS-R high	14.3 (12.8-15.8)	16.7 (15.1-18.3)	58	13 (10.8-15.2)	40	13.2 (11.1-15.4)	37	***0.0006***
p-value between	***<0.0001***	***<.0001***		***0.0019***		***0.0006***		
**IES Avoidance**		11.6 (10.4-12.7)	150	9.5 (8.2-10.9)	85	8 (6.8-9.2)	96	***<0.0001***
baseline PSWQ low	6.6 (5.2-7.9)	7.4 (5.8-9)	60	6.4 (4.3-8.5)	31	5.9 (4-7.8)	37	*0.3912*
baseline PSWQ high	11.8 (10.7-12.8)	14.3 (13-15.6)	90	11.6 (10-13.2)	54	9.3 (7.8-10.9)	59	***<.0001***
p-value between	***<0.0001***	***<.0001***		***0.0001***		***0.0056***		
baseline IUS-R low	7.5 (6.3-8.8)	8.7 (7.2-10.2)	77	7.5 (5.6-9.5)	39	6.3 (4.6-8)	47	*0.0624*
baseline IUS-R high	11.9 (10.7-13.1)	14.6 (13.1-16.1)	73	11.5 (9.7-13.3)	46	9.6 (8-11.3)	49	***<.0001***
p-value between	***<0.0001***	***<.0001***		***0.0032***		***0.0069***		

baseline PSWQ & IUS-R low	6.3 (4.8-7.8)	6.5 (4.7-8.3)	45	5.9 (3.5-8.3)	25	6.4 (4.2-8.6)	25	*0.8955*
baseline PSWQ high & baseline IUS-R low	9.5 (7.7-11.3)	11.8 (9.6-13.9)	32	10.4 (7.3-13.5)	14	6.5 (4.1-8.9)	22	***0.0011***
baseline PSWQ low & baseline IUS-R high	7.9 (5.3-10.5)	10.1 (6.9-13.2)	15	8.6 (3.9-13.3)	6	5 (1.8-8.3)	12	*0.038*
baseline PSWQ & IUS-R high	13 (11.7-14.2)	15.7 (14.1-17.3)	58	12.2 (10.4-14.1)	40	10.9 (9.1-12.8)	37	***<.0001***
p-value between	***<0.0001***	***<.0001***		***0.0008***		***0.0009***		
**IES Arousal**		7.6 (6.7-8.4)	150	6.2 (5.1-7.3)	85	5.7 (4.7-6.7)	96	***0.0012***
baseline PSWQ low	4.1 (3-5.3)	4.5 (3.2-5.7)	60	4 (2.3-5.7)	31	3.9 (2.3-5.4)	37	*0.7294*
baseline PSWQ high	8.1 (7.2-9)	9.6 (8.6-10.6)	90	7.7 (6.4-9.1)	54	7 (5.7-8.2)	59	***0.0002***
p-value between	***<0.0001***	***<.0001***		***0.001***		***0.0028***		
baseline IUS-R low	4.8 (3.8-5.9)	5.4 (4.3-6.6)	77	4.7 (3.1-6.3)	39	4.4 (3-5.8)	47	*0.3511*
baseline IUS-R high	8.2 (7.2-9.3)	9.8 (8.6-11)	73	7.8 (6.4-9.3)	46	7.1 (5.7-8.5)	49	***0.0004***
p-value between	***<0.0001***	***<.0001***		***0.0045***		***0.0086***		
baseline PSWQ & IUS-R low	4 (2.7-5.3)	4.1 (2.7-5.5)	45	3.8 (1.8-5.7)	25	4.2 (2.4-6.1)	25	*0.9025*
baseline PSWQ high & baseline IUS-R low	6.1 (4.5-7.6)	7.3 (5.6-8.9)	32	6.2 (3.6-8.7)	14	4.7 (2.7-6.7)	22	*0.0862*
baseline PSWQ low & baseline IUS-R high	4.6 (2.3-6.8)	5.5 (3.1-7.9)	15	5 (1.1-8.8)	6	3.2 (0.5-5.9)	12	*0.3384*
baseline PSWQ & IUS-R high	9.3 (8.2-10.3)	10.9 (9.7-12.1)	58	8.6 (7-10.2)	40	8.2 (6.7-9.7)	37	***0.0009***
p-value between	***<0.0001***	***<.0001***		***0.0021***		***0.0009***		
